# Establishment of Tools for Neurogenetic Analysis of Sexual Behavior in the Silkmoth, *Bombyx mori*

**DOI:** 10.1371/journal.pone.0113156

**Published:** 2014-11-14

**Authors:** Taketoshi Kiya, Koudai Morishita, Keiro Uchino, Masafumi Iwami, Hideki Sezutsu

**Affiliations:** 1 Division of Life Sciences, Graduate School of Natural Science and Technology, Kanazawa University, Kanazawa, Ishikawa, Japan; 2 Transgenic Silkworm Research Unit, National Institute of Agrobiological Sciences, Tsukuba, Ibaraki, Japan; Institute of Plant Physiology and Ecology, China

## Abstract

**Background:**

Silkmoth, *Bombyx mori*, is an ideal model insect for investigating the neural mechanisms underlying sex pheromone-induced innate behavior. Although transgenic techniques and the GAL4/UAS system are well established in the silkmoth, genetic tools useful for investigating brain function at the neural circuit level have been lacking.

**Results:**

In the present study, we established silkmoth strains in which we could visualize neural projections (UAS-mCD8GFP) and cell nucleus positions (UAS-GFP.nls), and manipulate neural excitability by thermal stimulation (UAS-dTrpA1). In these strains, neural projections and nucleus position were reliably labeled with green fluorescent protein in a GAL4-dependent manner. Further, the behavior of silkworm larvae and adults could be controlled by GAL4-dependent misexpression of dTrpA1. Ubiquitous dTrpA1 misexpression led both silkmoth larvae and adults to exhibit seizure-like phenotypes in a heat stimulation-dependent manner. Furthermore, dTrpA1 misexpression in the sex pheromone receptor neurons of male silkmoths allowed us to control male sexual behavior by changing the temperature. Thermally stimulated male silkmoths exhibited full sexual behavior, including wing-flapping, orientation, and attempted copulation, and precisely approached a thermal source in a manner similar to male silkmoths stimulated with the sex pheromone.

**Conclusion:**

These findings indicate that a thermogenetic approach using dTrpA1 is feasible in Lepidopteran insects and thermogenetic analysis of innate behavior is applicable in the silkmoth. These tools are essential for elucidating the relationships between neural circuits and function using neurogenetic methods.

## Introduction

Sex pheromones have an essential role in proper mate recognition and efficient partner identification [1], [2]. In general, sex pheromones comprise a mixture of chemicals and/or peptides, and function effectively when the ratios of their components are appropriate [1], [2]. The silkmoth, *Bombyx mori*, is an excellent model organism for sex pheromone identification and neural mechanisms of pheromone-induced innate behavior, because full sexual behavior can be induced in males by a single sex pheromone component, bombykol [2]–[8]. The female silkmoth emits a blend of two pheromone components, bombykol and bombykal, which have excitatory and inhibitory effects on male sexual behavior, respectively [9]–[11]. Bombykol receptors (*BmOR1*) are expressed only in the male antennae, and activation of *BmOR1*-expressing neurons is sufficient to induce sexual behavior [12], [13]. Taking advantage of the simple but robust silkmoth pheromone system and the relatively large size of the silkmoth brain, neural mechanisms of sex pheromone-induced sexual behavior have been extensively analyzed with electrophysiologic, optophysiologic, morphologic, and genetic methods [4]–[8], [14]–[25]. Recently, a novel molecular biologic technique using an immediate early gene, *Hr38*, as a neural activity marker revealed the distribution of cells activated by bombykol or bombykal exposure in the male silkmoth brain [26]. These studies led us to establish a neurogenetic method to visualize and manipulate a defined subset of neurons involved in sexual behavior in a reproducible manner and to elucidate the neural mechanisms with cellular resolution.

The silkmoth, *B. mori*, is Lepidopteran insect for which a transgenic technique is established [27]. In addition, establishment of the powerful GAL4/UAS system enabled us to conduct functional analyses of neural circuits at the molecular and cellular levels, like studies of the vinegar fly, *Drosophila melanogaster* [28]. Furthermore, the *piggyBac*-based enhancer trap system provides the opportunity to conduct non-biased genetic screening and reveal novel neural mechanisms that govern sexual behavior [29]. Despite the long history of studies and effective molecular genetic methods, tools useful for a neurogenetic approach in *B. mori* have been lacking.

A thermogenetic approach with a thermosensor protein dTrpA1 and temperature-sensitive mutant of dynamin, Shibire^ts1^, has been used successfully in *D. melanogaster* [30]–[35]. Recent progress in optogenetics allows us to control neural activity with high time-resolution [36]. Compared to the optogenetic approach, which is also applicable to silkmoth [25], the thermogenetic approach is less invasive and easier to apply. Neural activity or neural transmission can be activated or repressed simply by changing the culture temperature of the insects [30], [32]–[35]. In addition, thermogenetics can be used to control activity of neurons that reside deep inside the insect body. Because the silkmoth cuticle is thick and opaque, and the head is too small to be connected to optic stimulation fibers, establishing thermogenetic tools is essential for investigating neural circuit function on behavior under free-moving conditions.

In the present study, to establish methods for visualizing and manipulating a subset of neurons, we generated and analyzed silkmoth strains that expressed mCD8-fused green fluorescent protein (GFP), GFP.nls, dTRPA1, or Shibire^ts1^, under the control of GAL4. We demonstrated that mCD8GFP and GFP.nls localized to the plasma membrane and cell nucleus, respectively, indicating that these strains are useful for visualizing neural projection patterns and nucleus position. In addition, misexpression of dTrpA1, a thermosensor protein of *D. melanogaster* [30], enabled us to control silkmoth behavior in a thermal stimulation-dependent manner, providing evidence that thermogenetic neural activation is effective in silkmoth. In contrast, misexpression of Shibire^ts1^, a temperature-sensitive dynamin of *D. melanogaster* [31], was not effective in disturbing silkmoth behavior by changing the temperature. The present study paves the way for analysis of the neural mechanisms underlying the sexual behavior of silkmoths using a neurogenetic approach.

## Results

**Figure 1:**
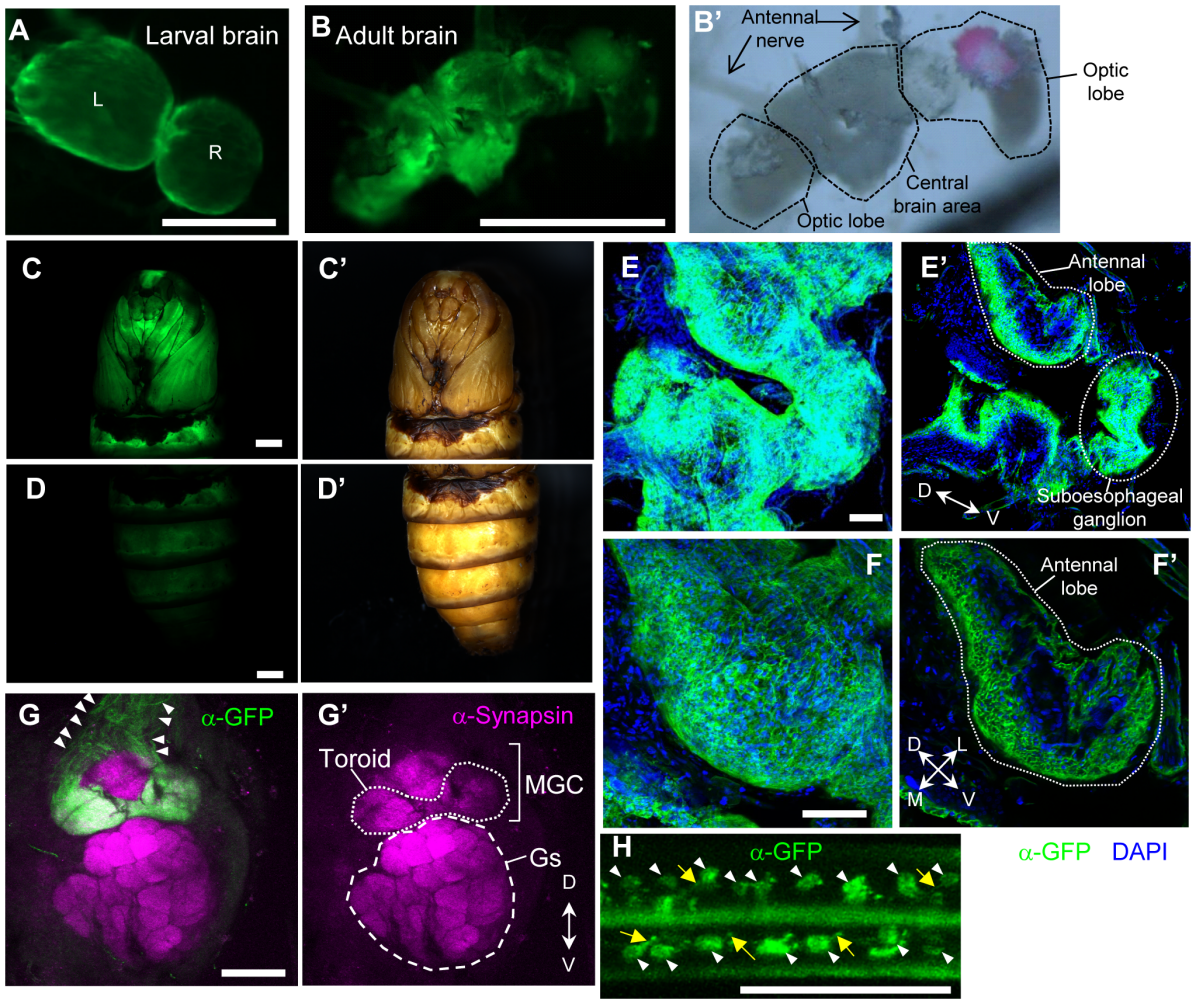
Visualization of cellular surface by mCD8GFP expression in silkmoths. (A–F) GFP fused with the membrane-tethering signal polypeptide (mCD8GFP) was ubiquitously expressed by crossing *UAS-mCD8GFP* and *Actin A3-GAL4* strains. GFP fluorescent images of the brains of second instar larva (A) and male adult moth (B), and pupal body (C, D). Brightfield images (B′–D′). Red color in the eyes is due to DeRed expression by the 3xP3 promoter used as a selection marker (B′, C′). L: Left brain hemisphere, R: Right brain hemisphere. (E, F) GFP expression was visualized with antibody staining (green). Nuclear DNA was visualized with DAPI (blue). Pictures of maximum intensity projection (E and F) or a single optical plane (E′ and F′) of confocal images of the whole brain (E and E′) and the antennal lobe (F and F′). Note that the GFP signal is detected at the cellular surface. (G, H) Visualization of sex pheromone receptor neurons by crossing *UAS-mCD8GFP* and *BmOR1-GAL4* strains. (G) GFP and glomerular structure of the antennal lobe was visualized with anti-GFP and anti-synapsin antibody, respectively. Each neural projection was visible (arrowhead) and axon terminals were correctly targeted to the bombykol-responsive glomeruli (toroid of the macroglomerular complex [MGC]). Gs: Ordinary glomeruli. (H) Each receptor cell (arrowhead) and neurite (arrow) was visible. Scale bars: 100 µm (A, E–H), 1 mm (B–D).

Visualization of the projection pattern of neurons is essential for revealing functional neural connections. For this purpose, we generated a silkmoth strain that can express membrane-tethering GFP [mCD8GFP: mouse T cell receptor (CD8)-fused GFP] under the control of GAL4 (UAS-mCD8GFP strain) [37]. Using a GAL4 driver strain that has ubiquitous GAL4 expression (*Actin A3-GAL4*) [38], we expressed mCD8GFP throughout the body and analyzed its expression pattern. Fluorescent signals were detected from embryos to adult moths and were visible on the brain and body surfaces under epifluorescent microscopy (Figure 1A–D). Antibody staining of brains confirmed that GFP localized to the cellular membrane (Figure 1E–F). We also examined mCD8GFP expression in the sex pheromone (bombykol) receptor neurons using the *BmOR1-GAL4* strain [13]. *BmOR1* is the bombykol receptor gene expressed only in the male antennae, and the *BmOR1-GAL4* strain expresses transgenes in almost all (96.8%) *BmOR1*-expressing cells [12], [13], [25]. Anti-GFP staining revealed that *BmOR1*-expressing neurons specifically project to the toroid of the macroglomerular complex of the antennal lobe, which is the bombykol-responsive glomerulus (Figure 1G) [6]. Each projecting axon was visible in this strain (arrowheads in Figure 1G). In the antenna, each *BmOR1*-expressing neuron and its neurite were also visible by anti-GFP staining (Figure 1H). These findings indicate that this strain is useful for visualizing the neural projection pattern and performing detailed analyses of neural connections.

**Figure 2:**
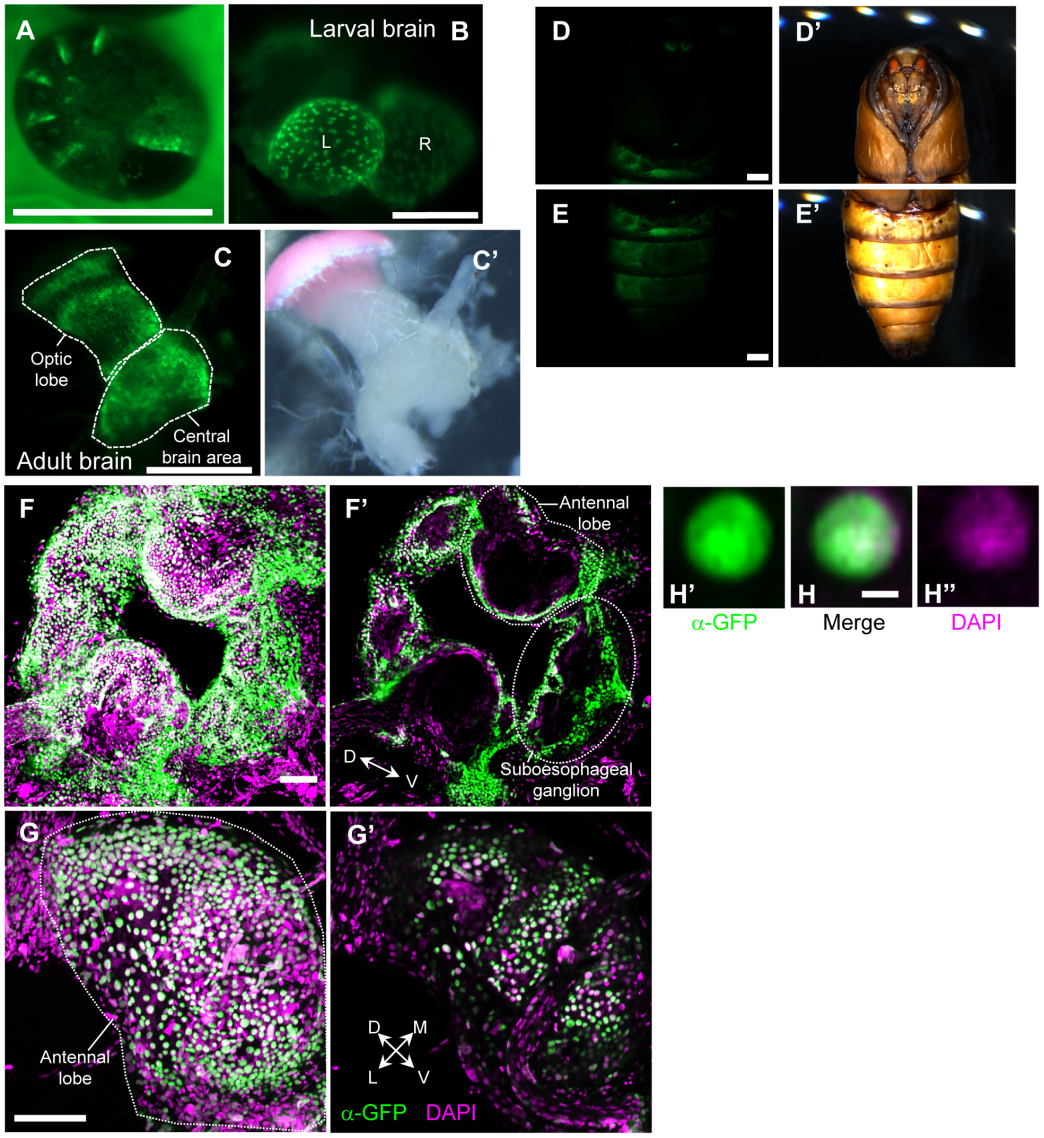
Visualization of cellular position by nuclear-localizing GFP expression. GFP fused with the nuclear localization signal (GFP.nls) was ubiquitously expressed by crossing *Actin A3-GAL4* and *UAS-GFP.nls* strains. (A–E) GFP fluorescent images of embryo (A), the brains of second instar larva (B) and male adult moth (C), and pupal body (D, E). Brightfield images (C′–E′). (F–H) GFP expression was visualized with antibody staining (green). Nuclear DNA was visualized with DAPI (magenta). Pictures of maximum intensity projection (F, G) or a single plane (F′, G′, H) of confocal images of the whole brain (F, F′) and the antennal lobe (G, G′). (H) Because GFP localizes exclusively to the nuclei, the cellular position is identifiable. Scale bars: 1 mm (A, D, E), 100 µm (B, F, G), 500 µm (C), 5 µm (H).

In addition to visualizing the neural projections, it is essential to establish methods for identifying the cellular position and quantifying cell number. For this purpose, we generated a strain that expressed GFP fused with nuclear localization signal (GFP.nls) under the control of GAL4 (UAS-GFP.nls strain). Ubiquitous expression of GFP.nls with *Actin A3-GAL4* resulted in dot-like fluorescent signals throughout the body from embryo to adult (Figure 2A–E). Antibody staining also confirmed that the GFP localized exclusively to the nuclei (Figure 2 F–H), and thus allowed for identification of GFP-positive cell positions due to the segregated GFP signals among cells. The ability to quantify GFP-positive cells could be useful in future studies.

**Figure 3:**
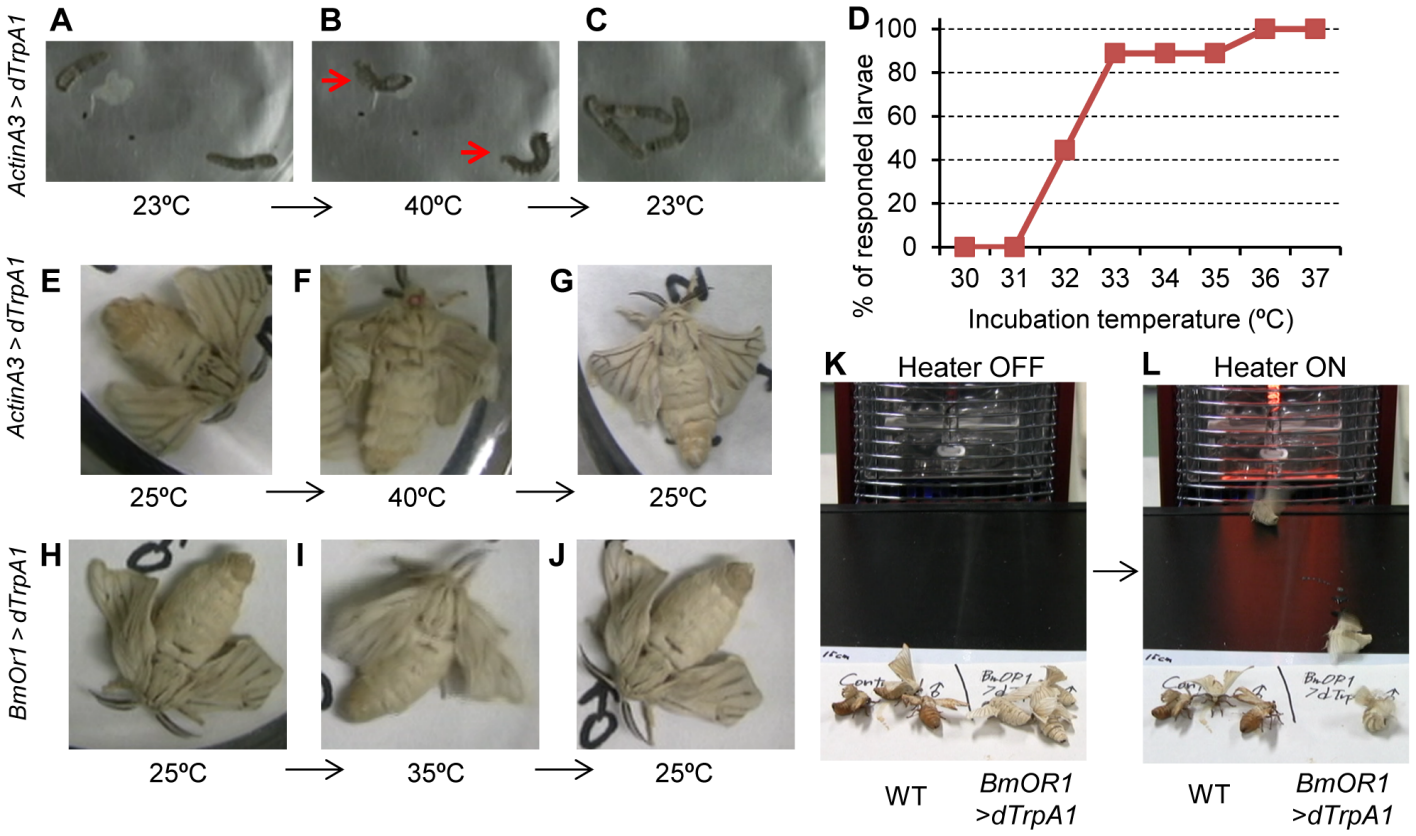
Control of silkmoth behavior by the thermogenetic approach. (A–G) The thermosensor protein, dTrpA1, was ubiquitously expressed by crossing *Actin A3-GAL4* and *UAS-dTrpA1* strains. (A–C) Thermal stimulation induced systemic contraction in second instar larvae. Phenotypes were analyzed at 23°C and 40°C. The convulsion-like phenotype was observed at 40°C (B) and was reversible by decreasing the temperature to 23°C (C) (Video S1). (D) Cumulative curve of responsive larvae to incubation temperature. Vertical axis indicates ratio of larvae showing contractile phenotype during the observation time (10 min for each temperature; N = 9). (E–G) A convulsion-like phenotype was induced in male adult moths at 40°C (F), and was reversible at 25°C (G) (Video S2). (H–J) Sexual behavior was reversibly controlled by thermal stimulation in the male moths with dTrpA1 misexpression in the bombykol receptor neurons (Video S3). (K, L) Thermogenetic activation of the bombykol receptor neurons induced pheromone orientation behavior. When the thermal source (heater) was turned on, male transgenic moths began wing-flapping, orienting to the heat source, and attempting copulation, and approached the heat source (Video S4).

To investigate the functional roles of a defined set of neurons in behavior, tools that can selectively activate or repress neural activity or transmission in a temporary-regulated manner are needed. Therefore, we generated the UAS-dTrpA1 strain expressing dTrpA1 under the control of GAL4 and evaluated the usefulness of dTrpA1 as a thermogenetic tool in silkmoth. We selected dTrpA1 as a thermogenetic tool because the channel pore of dTrpA1 begins to open at approximately 25°C, which is compatible with silkmoth rearing. First, using second instar larvae with ubiquitous misexpression of dTrpA1, we investigated the behavioral response of these larvae to thermal stimulation (Figure 3A–C and Video S1). In this assay, we used a thermal cycler as a simple thermal stimulator and changed the larval ambient temperature from 23°C (permissive temperature) to 40°C (test temperature). The larvae exhibited systemic contraction and became C-shaped upon the shift in temperature, probably due to strong contraction of the body muscles. This phenotype was reversible and all larvae behaved normally after the temperature was returned to 23°C. In addition, this phenotype was observed only in the larvae that possessed both *Actin A3*-GAL4 and UAS-dTrpA1 genes (Video S1). These findings indicate that dTrpA1 effectively functions as a thermosensor channel, even under heterologous conditions in silkmoth cells. To accurately measure the behavioral threshold temperature of dTrpA1-expressing larvae, we precisely controlled the ambient temperature using a heat block to increase the temperature from 30°C to 37°C in 1°C steps every 10 min, and observed the larval responses (Figure 3D). Because preliminary experiments revealed that transgenic larvae begin to show the temperature-sensitive phenotype between 30°C to 35°C, we began this analysis at 30°C. Approximately half the larvae showed a contractile response at 32°C and almost all larvae (89%; 8 out of 9 larvae) showed a response at 33°C during a 10-min observation time at each temperature, indicating a behavioral threshold temperature of 32°C.

We also addressed whether the thermogenetic approach works in adult moths. In adults, the seizure-like phenotype was observed only when moths were incubated at higher temperatures than larvae (Figure 3E–G and Video S2). Effective stimulation of adult male moths required incubation at 40°C (65%; 15 out of 23 moths responded). The difference in threshold temperatures between larvae and adult moths might be due to varying levels of dTrpA1 expression in the body muscles at different developmental stages. These thermal stimulation-induced behavioral phenotypes were not observed in control strains possessing only *Actin A3*-GAL4 or UAS-dTrpA1 (0 out of 8 moths responded, each). To further evaluate the usefulness of the UAS-dTrpA1 strain, we examined the effects of dTrpA1 misexpression in *BmOR1*-expressing cells and tested whether we could control the sexual behavior by thermal stimulation. In the silkmoth, *BmOR1* is expressed only in the male antennae, and activation of *BmOR1*-expressing neurons is sufficient to induce full sexual behavior [12], [13], [25]. Within 1 min of incubation at 35°C, all responsive male moths exhibited sexual behavior with vigorous wing-flapping and attempted copulation (Figure 3H–J and Video S3) (61%; 22 out of 36 moths responded). Incubation at higher temperature (40°C) did not increase responsive rate. These results indicated that 35°C is sufficient to stimulate the antennal cells, suggesting that there are cell-type and/or tissue-type differences in the threshold temperature. Surgical removal of bilateral antennae from the base with a fine scissor (N = 5) completely abolished the thermal stimulation-dependent sexual behavior (0 out of 5 moths responded), confirming the specificity of dTrpA1 function in the antennae. In addition, no thermal stimulation-induced sexual behavior was observed in control strains possessing only *BmOR1*-GAL4 or UAS-dTrpA1 (0 out of 3 moths or 0 out of 18 moths responded, respectively).

Finally, we examined whether a thermally-guided silkmoth would precisely approach a thermal source similar to the innate pheromone orientation behavior to female moths (Figure 3K, L, and Video S4). Male silkmoths with misexpressed dTrpA1 in *BmOR1*-expressing cells started female searching behavior upon thermal stimulation (90%; 9 out of 10 moths showed behavior) and reached the heat source (100%; 9 out of 9 moths reached the heater). In contrast, control moths (wild-type) did not show apparent sexual behavior in response to thermal stimulation (0 out of 3 moths). These findings indicate that the thermogenetic approach using dTrpA1 is effective for controlling innate sexual behavior in silkmoths.

We also generated UAS-Shibire^ts1^ expressing temperature-sensitive mutant dynamin under the control of GAL4. Despite thorough analyses, we observed no detectable phenotypes by ubiquitous or bombykol receptor cell-selective Shibire^ts1^ expression (0 out of 13 moths or 0 out of 16 moths, respectively). Because the protein sequence of dynamin is largely different between the fly, *D. melanogaster*, and the silkmoth, *B. mori*, Shibire^ts1^ misexpression may have only a weak dominant-negative effect.

## Discussion

In the present study, we established useful silkmoth strains for visualizing neural projection patterns and cell nucleus positions, and regulating neural activity by thermal stimulation. The silkmoth, *B. mori*, has long been used for sex pheromone studies: identification of sex pheromone compounds [9]–[11], regulatory mechanisms of pheromone biosynthesizing pathways [39], and neural mechanisms of sex pheromone recognition and pheromone-induced sexual behavior [3]–[8], [13], [23], [25]. Neurogenetic methods for investigating functional relationships between neural circuits and behavior, which can only be studied in living animals, however, have been lacking. Therefore, the strains for neurogenetics established in the present study will be powerful tools for revealing brain function at the circuit and/or cellular levels in future studies, as discussed below.

First, in the present study, expression of GFP fused with plasma membrane-tethering signal (mCD8GFP) and nuclear localization signal (GFP.nls) in the silkmoth brain successfully targeted the cellular membrane and nuclei, respectively. Each neurite in the antenna and brain could be identified when mCD8GFP was expressed in bombykol receptor cells. In addition, each nucleus could be identified, even when GFP.nls was ubiquitously expressed in the brain. Therefore, these strains are highly useful for revealing neural projection patterns, cellular position, and cell numbers of a defined set of neurons, in combination with promoter GAL4 strains and enhancer trap GAL4 strains.

Second, we demonstrated that the thermogenetic approach using dTrpA1 is effective in silkmoth. To our knowledge, this is the first report showing that ectopically expressed dTrpA1 is effective *in vivo* in insects other than *D. melanogaster*. Because thermogenetic methods are non-invasive and easy to use for stimulating deep brain cells, the UAS-dTrpA1 strain will be an essential tool for investigating causal links between neural circuits and behavior under free-moving conditions. Functional genetic screening using dTrpA1 recently led to the identification of neural circuits that regulate innate behaviors like sexual behavior and aggressive behavior, as well as stereotypic behavior, such as walking direction, in *D. melanogaster* [32]–[35]. Because a *piggyBac*-based genetic screen is also possible in silkmoth [29], our findings provide opportunities for conducting a functional genetic screen and identifying the neural circuits that underlie sexual behavior. In the male silkmoth, activation of bombykol receptor cells is sufficient to induce all steps of sexual behavior, as reported previously [13], [25] and confirmed in the present study. In addition, adult moths do not show any behavior other than sexual behavior. Utilizing these simple and advantageous characteristics, we expect future genetic studies will identify the essential neural circuits and components underlying sexual behavior.

Third, the threshold temperature for behavior induction was 32∼40°C in silkmoths with dTrpA1 misexpression, in contrast with *D. melanogaster*, in which the threshold temperature for behavior induction is 27∼29°C [32]–[35]. A possible explanation for this discrepancy is a species-specific difference in the cellular environment between fly and silkmoth. The molecular mechanisms that determine the temperature threshold of channel-gating remain obscure in dTrpA1, but gating properties of thermal TRP channels are known to be modified by external ligands, such as capsaicin (TRPV1) and menthol (TRPM8) [40]. Therefore, it is possible that the molecular environment surrounding dTrpA1 channels influences the gating properties in silkmoth cells. This shift in the threshold temperature allowed us to rear dTrpA1-expressing silkmoth larvae under normal temperature conditions (25°C), eliminating the side effects and developmental delays induced by low temperature rearing. Furthermore, the threshold temperature for behavioral induction differed between developmental stages, as well as between tissues. These differences could derive from differences in the GAL4 expression levels, which would result in differences in the dTrpA1 expression levels between developmental stages and between tissues. Another possibility is that the molecular environment surrounding the dTrpA1 channels differs between developmental stages and between tissues. In future studies using other GAL4 drivers for activating a subset of cells using dTrpA1, careful examination of activation temperature is needed.

Recently, the *Bombyx* ortholog of the TrpA1 channel (BmTrpA1) was reported to open at temperatures above 21°C and to function as a thermosensor to induce the transgenerational diapause phenotype [41]. The molecular mechanisms that determine the properties of temperature-dependent channel-gating and the species differences that match the life history and habitat of each insect remain to be elucidated in future studies.

In conclusion, the strains established in the present study will be essential tools for investigating the relationships between neural circuits and behavior in silkmoths. These strains will help to elucidate the neural basis of sexual behavior in silkmoth.

## Materials and Methods

### Silkmoth strains

The non-diapausing *B. mori* strain, *w1-pnd*, was used to generate transgenic silkmoths. The generated transgenic silkmoths were crossed to diapausing strain *white*/c for several generations and maintained. Larvae were reared on an artificial diet (Nihon Nosan Kogyo, Yokohama, Japan) at 25°C under a 12-h light/12-h dark photoperiod cycle. In the initial experiments using larvae and silkmoths that ubiquitously express dTrpA1, larvae and adults were reared and maintained at 18°C until use. After confirming that the activation threshold of dTrpA1 in silkmoth was greater than 30°C, larvae were reared at 25°C. Adult moths were used within 0 to 4 days after eclosion.

### Generation of transgenic silkmoths

*pBacMCS-UAS-SV40* was generated by subcloning a *Bgl*II-*Apa*I fragment containing the *SV40 terminator* amplified by the polymerase chain reaction using the primers (forward: 5′-GCAGATCTTCAGCCATACCACATTTGTAGA-3′ and reverse: 5′-GCGGGCCCTGAGTTTGGACAAACCACAACT-3′) from *pBac-UAS-SV40/3xP3EGFP* into *pBacMCS-UAS* [42]. For the *UAS-mCD8GFP*, *UAS-GFP.nls*, and *UAS-dTrpA1* constructs, *Not*I*-Not*I polymerase chain reaction fragments containing *mCD8GFP*, *GFP.nls*, and *dTrpA1* were subcloned immediately downstream from the *UAS* of *pBacMCS-UAS-SV40*. The *Asc*I-*Asc*I fragment containing the *fibroin L chain* (*FibL*) promoter, *EGFP*, and *FibL 3′UTR* amplified by polymerase chain reaction using the primers (forward: 5′-GCGGCGCGCCGGTACGGTTCGTAAAGTTCA-3′ and reverse: 5′-GCGGCGCGCCTATATGGTATTATCGAATAC-3′) was subcloned into these constructs to generate *pBac[UAS-mCD8GFP-SV40]*, *pBac[UAS-GFPnls-SV40]*, and *pBac[UAS-dTrpA1-SV40]*, respectively (Figures S1, S2). *pUAST-mCD8GFP* [37] and *D. melanogaster* carrying *UAS-GFP.nls* and *UAS-dTrpA1* (Bloomington Drosophila Stock Center, Bloomington, IN) were used as gene sources. Transgenic silkmoths were generated by the *piggyBac*-mediated germ-line transformation methods, as described previously [27]. At least three independent lines were generated and analyzed for each strain.

### Immunohistochemistry

The brains of male and female silkmoths were dissected and fixed in 4% paraformaldehyde/phosphate-buffered saline overnight at 4°C. The brains were washed three times in phosphate-buffered saline containing 0.3% TritonX-100 (PBTX), blocked in 7% normal donkey serum in PBTX for 3 h at room temperature, and incubated in rabbit anti-GFP antibody (1/200; Clontech, Mountain View, CA), anti-synapsin monoclonal antibody (1/100; Developmental Studies Hybridoma Bank, Iowa City, IA), and 1% normal donkey serum for 1 week at 4°C. After several washes in PBTX, signals were developed by incubation with fluorescein isothiocyanate-conjugated anti-rabbit IgG (1/200; Cappel, Aurora, OH) and TexasRed-conjugated anti-mouse IgG (1/200; Cappel) for two overnights at 4°C. The nuclear DNA was stained with 4′,6-diamidino-2-phenylindole (DAPI) and pictures were obtained using the confocal microscope LSM5 (Carl Zeiss, Germany).

## Supporting Information

Figure S1 **Flowchart of** ***piggyBac*** **vector construction.** Example of *pBac[UAS-dTrp-SV40]* vector construction. Other vectors were constructed in this same manner.
(TIF)

Figure S2 **Schematic diagrams of** ***piggyBac*** **vectors used to generate transgenic silkmoths.**
(TIF)

Video S1 **Behavioral response of larvae to thermal stimulation.** Effectiveness of thermogenetics using dTrpA1 was verified using second instar larvae. Larvae possessing both *Actin A3*-GAL4 and UAS-dTrpA1 (top left), only *Actin A3*-GAL4 (top right), only UAS-dTrpA1 (bottom left), and no transgenes (bottom right), were analyzed. The ambient temperature was changed from 23°C to 40°C.
(MP4)

Video S2 **Behavioral response of adult male moths to thermal stimulation.** Effectiveness of ubiquitous dTrpA1 misexpression in male silkmoths was analyzed. This movie shows only males possessing both *Actin A3*-GAL4 and UAS-dTrpA1.
(MP4)

Video S3 **Thermogenetic activation of bombykol receptor cells was sufficient to induce courtship behavior.** Male moths with dTrpA1 misexpression in the bombykol receptor cells were warmed at 35°C. Thermogenetic activation was sufficient for reversible induction of courtship behavior.
(MP4)

Video S4 **Thermally-guided transgenic silkmoths can precisely approach a thermal source.** To examine whether thermogenetic activation of sex pheromone receptor cells is sufficient to induce female searching behavior, wild-type males (control: left) and transgenic males (*BmOR1*>*dTrpA1*: right) were stimulated with a carbon heater. Upon turning on the heater, transgenic moths exhibited courtship behavior and approached the heater.
(MP4)
