# Supplementary figures and images for: Establishment of Tools for Neurogenetic Analysis of Sexual Behavior in the Silkmoth, Bombyx mori
Source: PLoS One. 2014 Nov 14;9(11):e113156. doi: 10.1371/journal.pone.0113156 (PMC4232604; doi:10.1371/journal.pone.0113156)

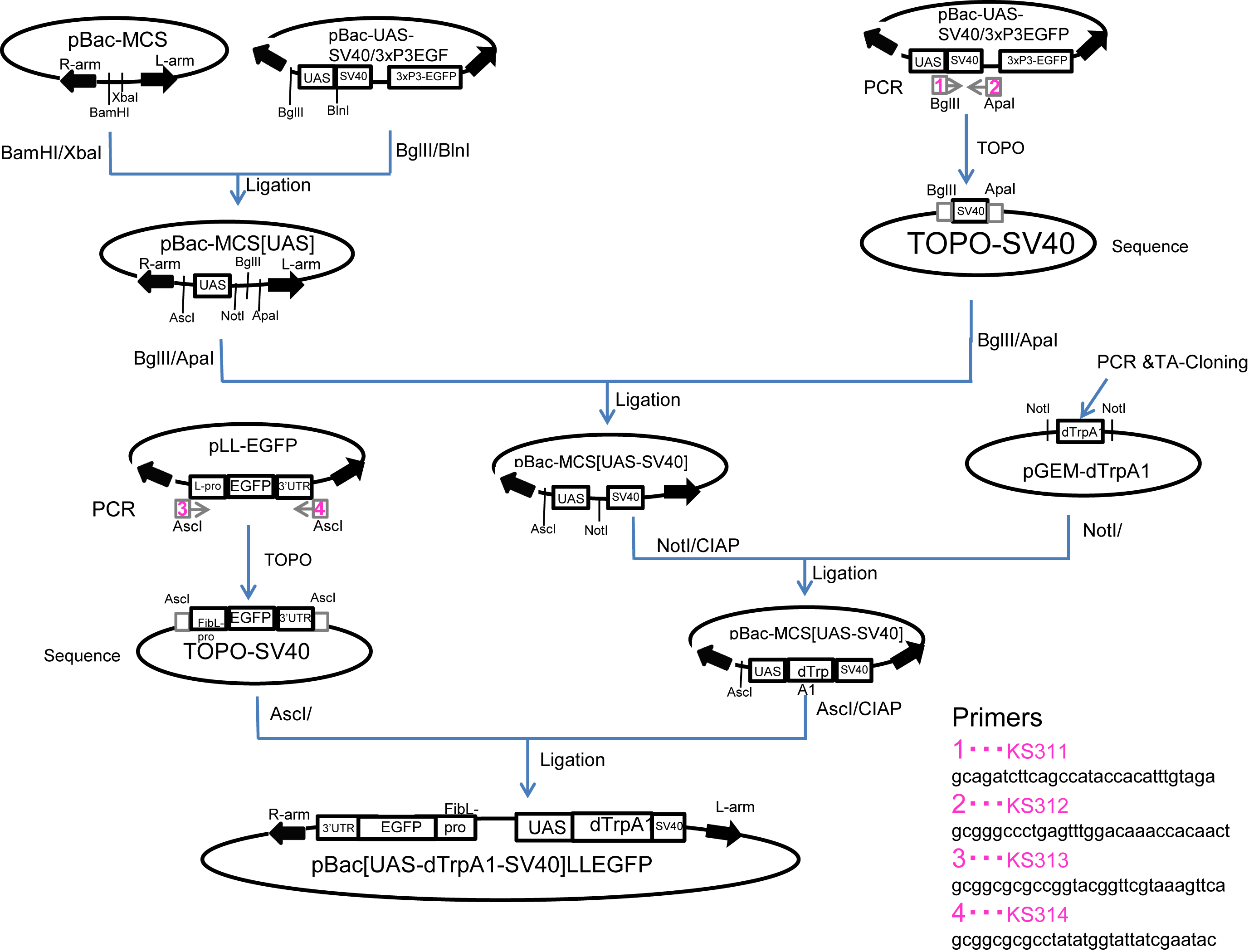

Supplement: Figure S1 — Flowchart of piggyBac vector construction. Example of pBac[UAS-dTrp-SV40] vector construction. Other vectors were constructed in this same manner. (TIF) [file pone.0113156.s001.tif]

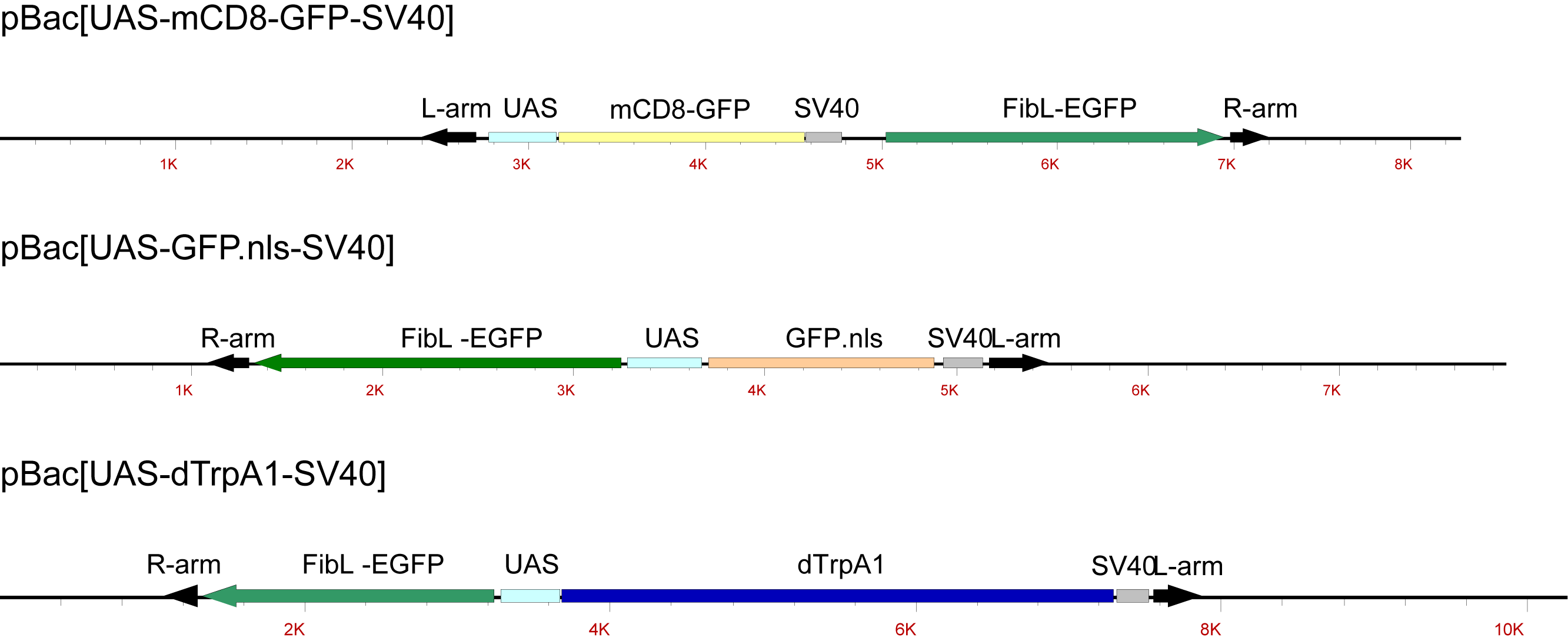

Supplement: Figure S2 — Schematic diagrams of piggyBac vectors used to generate transgenic silkmoths. (TIF) [file pone.0113156.s002.tif]
